# Publisher Correction: Cardiac disruption of SDHAF4-mediated mitochondrial complex II assembly promotes dilated cardiomyopathy

**DOI:** 10.1038/s41467-022-32492-w

**Published:** 2022-08-10

**Authors:** Xueqiang Wang, Xing Zhang, Ke Cao, Mengqi Zeng, Xuyang Fu, Adi Zheng, Feng Zhang, Feng Gao, Xuan Zou, Hao Li, Min Li, Weiqiang Lv, Jie Xu, Jiangang Long, Weijin Zang, Jinghai Chen, Feng Gao, Jian Ding, Jiankang Liu, Zhihui Feng

**Affiliations:** 1 School of Health and Life Sciences, University of Health and Rehabilitation Sciences, 266071 Qingdao, Shandong China; 2 Center for Mitochondrial Biology and Medicine, The Key Laboratory of Biomedical Information Engineering of Ministry of Education, School of Life Science and Technology, Xi’an Jiaotong University, 710049 Xi’an, Shaanxi China; 3 Key Laboratory of Aerospace Medicine of the Ministry of Education, School of Aerospace Medicine, Fourth Military Medical University, 710032 Xi’an, China; 4 Department of Cardiology, Provincial Key Lab of Cardiovascular Research, Second Affiliated Hospital, Zhejiang University School of Medicine, 310009 Hangzhou, China; 5 Institute of Translational Medicine, Zhejiang University, School of Medicine, 310029 Hangzhou, China; 6 National & Local Joint Engineering Research Center of Biodiagnosis and Biotherapy, The Second Affiliated Hospital of Xi’an Jiaotong University, 710004 Xi’an, Shaanxi China; 7 Department of Pharmacology, School of Basic Medical Sciences, Xi’an Jiaotong University Health Science Center, Xi’an, Shaanxi China; 8 Frontier Institute of Science and Technology, Xi’an Jiaotong University, 710049 Xi’an, China

**Keywords:** Heart failure, Metabolism

Correction to: *Nature Communications* 10.1038/s41467-022-31548-1, published online 08 July 2022

The original version of this Article omitted from the author list the 17th author Feng Gao, who is from the Key Laboratory of Aerospace Medicine of the Ministry of Education, School of Aerospace Medicine, Fourth Military Medical University, Xi’an, 710032, China. This has been corrected in both the PDF and HTML versions of the Article.

