# Supplementary material for: Cardiac disruption of SDHAF4-mediated mitochondrial complex II assembly promotes dilated cardiomyopathy
Source: Nat Commun. 2022 Jul 8;13:3947. doi: 10.1038/s41467-022-31548-1 (PMC9270418; doi:10.1038/s41467-022-31548-1)
Supplement: Supplementary file 2 — Reporting Summary [file 41467_2022_31548_MOESM2_ESM.pdf]

nature portfolio

Corresponding author(s): Jiankang Liu; Jian Ding; Zhihui Feng

Last updated by author(s): Oct 7, 2021

Reporting Summary

Nature Portfolio wishes to improve the reproducibility of the work that we publish. This form provides structure for consistency and transparency in reporting. For further information on Nature Portfolio policies, see our Editorial Policies and the Editorial Policy Checklist.

Statistics

For all statistical analyses, confirm that the following items are present in the figure legend, table legend, main text, or Methods section.

n/a	Confirmed	
☐	☒	The exact sample size (n) for each experimental group/condition, given as a discrete number and unit of measurement
☐	☒	A statement on whether measurements were taken from distinct samples or whether the same sample was measured repeatedly
☐	☒	The statistical test(s) used AND whether they are one- or two-sided<br>*Only common tests should be described solely by name; describe more complex techniques in the Methods section.*
☒	☐	A description of all covariates tested
☒	☐	A description of any assumptions or corrections, such as tests of normality and adjustment for multiple comparisons
☐	☒	A full description of the statistical parameters including central tendency (e.g. means) or other basic estimates (e.g. regression coefficient) AND variation (e.g. standard deviation) or associated estimates of uncertainty (e.g. confidence intervals)
☐	☒	For null hypothesis testing, the test statistic (e.g. F, t, r) with confidence intervals, effect sizes, degrees of freedom and P value noted<br>*Give P values as exact values whenever suitable.*
☒	☐	For Bayesian analysis, information on the choice of priors and Markov chain Monte Carlo settings
☒	☐	For hierarchical and complex designs, identification of the appropriate level for tests and full reporting of outcomes
☐	☒	Estimates of effect sizes (e.g. Cohen's d, Pearson's r), indicating how they were calculated

Our web collection on statistics for biologists contains articles on many of the points above.

Software and code

Policy information about availability of computer code

Data collection	The data of confocal microscopy were collected using ZEN 2012 blue edition (ZEISS, Jena, Germany). Metabolomics GC-MS full spectrum analysis automatic metabolite identification and statistical analysis was used XploreMET 2.0 software (Metabo-Profile, Shanghai, China)
Data analysis	Statistical analysis were performed with GraphPad Prism GraphPad Prism software (V5 & V7). Metabolomics GC-MS full spectrum analysis automatic metabolite identification and statistical analysis was used XploreMET 2.0 software (Metabo-Profile, Shanghai, China). The strips of western blots were analyzed using Clinx chemi analysis (Clinx, Shanghai, China, Version2.1.1.8).

For manuscripts utilizing custom algorithms or software that are central to the research but not yet described in published literature, software must be made available to editors and reviewers. We strongly encourage code deposition in a community repository (e.g. GitHub). See the Nature Portfolio guidelines for submitting code & software for further information.

Data

Policy information about availability of data

All manuscripts must include a data availability statement. This statement should provide the following information, where applicable:

- Accession codes, unique identifiers, or web links for publicly available datasets
- A description of any restrictions on data availability
- For clinical datasets or third party data, please ensure that the statement adheres to our policy

RNA-Seq data generated during the study have been deposited in NCBI Gene Expression Omnibus (accession number GSE 163809). The data that support the finding of this study are available from NCBI Gene Expression Omnibus (accession number GSE 135055). Source data and other supporting informations are provided with this paper.

Field-specific reporting

Please select the one below that is the best fit for your research. If you are not sure, read the appropriate sections before making your selection.

- [x] Life sciences
- [ ] Behavioural & social sciences
- [ ] Ecological, evolutionary & environmental sciences

For a reference copy of the document with all sections, see nature.com/documents/nr-reporting-summary-flat.pdf

Life sciences study design

All studies must disclose on these points even when the disclosure is negative.

Sample size	Sample sizes were not pre-determined based on statistical analysis. We were based on our previous experience with relevant publications cited in the manuscript such as Wu S, et al. (Circulation 139, 1913-1936). For animal studies which usually cause the high variability in commonly, we typically used n≥6; For assays in which variability is commonly low, we typically used n<6. Experimental approach, availability and feasibility required to obtain statistically significant results were also taken in consideration.
Data exclusions	No data were exclued.
Replication	For each animal experiment, at least three volumes were analyzed per animal, and at least 3 animals were used per condition. For each cellular experiment, at least three independent trials were performed per condition. Replication of experiments was successful.
Randomization	All the mice were randomly selected and/or randomly grouped. For in vitro experiments, samples were randomly allocated to each group, and subsequently handled and processed identically.
Blinding	The investigators who performed the echocardiography analysis and cardiac injury surgeries were blinded to group allocation. Investigators were blinded during tissue collection and processing. The investigators who performed the biochemical analysis of the tissue were only informed of the numbering, not the group allocation. Similar procedures applied in cell experiments.

Reporting for specific materials, systems and methods

We require information from authors about some types of materials, experimental systems and methods used in many studies. Here, indicate whether each material, system or method listed is relevant to your study. If you are not sure if a list item applies to your research, read the appropriate section before selecting a response.

Materials & experimental systems

n/a	Involved in the study	
[ ]	[x]	Antibodies
[ ]	[x]	Eukaryotic cell lines
[x]	[ ]	Palaeontology and archaeology
[ ]	[x]	Animals and other organisms
[x]	[ ]	Human research participants
[x]	[ ]	Clinical data
[x]	[ ]	Dual use research of concern

Methods

n/a	Involved in the study	
[x]	[ ]	ChIP-seq
[x]	[ ]	Flow cytometry
[x]	[ ]	MRI-based neuroimaging

Antibodies

Antibodies used	The antibodies used were as follows:<br>GAPDH (Cell signaling, Cat. #5174s, 1:2000); VDAC (Cell signaling, Cat#4866, 1:2000); TFAM (Cell signaling, Cat#8706, 1:2000); CS (Cell signaling, Cat#14309, 1:2000); ACO2 (Cell signaling, Cat#6571, 1:2000); IDH2 (Cell signaling, Cat#56439, 1:2000); OGDH (Cell signaling, Cat#26865, 1:2000); SCS (Cell signaling, Cat#8071s, 1:2000); FH (Cell signaling, Cat#4567s, 1:2000); MDH2 (Cell signaling, Cat#11908, 1:2000); p-DRP1 (Ser616) (Cell signaling, Cat.#3455, 1:2000); p-DRP1(Ser637) (Cell signaling, Cat.#4867, 1:2000); DRP1 (Cell signaling, Cat.#8570, 1:2000); LC3B(Cell signaling, Cat.#3868, 1:2000); ERK (Cell signaling, Cat.#4695, 1:2000); p-Erk (Cell signaling, Cat.#4370, 1:2000); OPA1 (Cell signaling, Cat.#80471s, 1:2000); SDHA (Cell signaling, Cat.#11998s, 1:2000); AMPK (Cell signaling, Cat.#5832, 1:2000); p-AMPK (Cell signaling, Cat.#8208, 1:2000); SDHAF2 (Cell signaling, Cat.#45849, 1:2000); Histone H3 (Cell signaling, Cat.#4499, 1:2000); SDHB (Abcam, Cat.#178423, 1:2000); SDHAF1 (Abcam, Cat.#185222, 1:2000); SDHAF4 (Abcam, Cat.# 122196, 1:2000); SDHC (Abcam, Cat.#155999, 1:2000); SDHD (Abcam, Cat.#189945, 1:2000); FIS1 (Abcam, Cat.#71498, 1:2000); SDHAF3 (Novus, Cat.#NBP2-14259, 1:2000); HIF1α (Novus, Cat.# NB100-105, 1:2000); PGC-1α (Novus, Cat.# NBP1-04676, 1:2000); H3K4me1 (Active motif, Cat.# 39635, 1:1000); H3K27me2 (Active motif, Cat.#39920, 1:1000); H3K9me2 (Active motif, Cat.#39041, 1:1000); H3K9me3 (Active motif, Cat.#61013, 1:1000); Ubiquitin (Santa Cruz, Cat.#8017, 1:1000); MFN1 (Santa Cruz, Cat.#166644, 1:1000); MFN2 (Santa Cruz, Cat.#515647, 1:1000); SQSTM1 (Santa Cruz, Cat.#8359, 1:1000); NDUFA9 (Invitrogen, Cat.#PA5-36993, 1:2000); UQCRC1 (Invitrogen, Cat.#459140, 1:2000); COX4 (Invitrogen, Cat.#MA5-15078, 1:2000); ATP5A (Invitrogen, Cat.#459240, 1:2000); Anti-mouse IgG (H+L), F(ab')2 Fragment (Alexa Fluor® 488 Conjugate) (Cell signaling, Cat.#4408, 1:200); Anti-rabbit IgG (H+L), F(ab')2 Fragment (Alexa Fluor® 555 Conjugate) (Cell signaling, Cat.#4413, 1:200).

Validation	All antibodies were purchased from commercial sources and have been validated in previously published studies, e.g. Nature (PMID: 32494005) and J Clin Invest (PMID:34591791). All validation statements, citations, antibody details can be found on manufacturer's website. Positive and negative controls were also adopted for validation of antibody data, and several dilutions were tested for each antibody. For instance, the anti-SDHAF4 antibody was validated using SDHAF4-CKO mice.<br>Cell Signaling Technology validation statement: Western blotting remains one of the most common scientific methods for monitoring protein expression in cells or tissue. The accuracy of western blot results relies heavily of the quality of the primary antibody employed in the immunoblotting. Cell Signaling Technology (CST) provides the highest quality primary and secondary antibodiesavailable for western blotting. CST™ antibodies are produced in-house and validated extensively according to a rigorous protocol.<br>Abcam validation statement: Antibodies are validated in western blot using lysates from cells or tissues that we have identified to express the protein of interest. Once we have determined the right lysates to use, western blots are run and the band size is checked for the expected molecular weight. We will always run several controls in the same western blot experiment, including positive lysate and negative lysate. When possible, we also include knock-out (KO) cell lines as a true negative control for our western blots. We arealways increasing the number of KO-validated antibodies we provide. In addition, we run old stock alongside our new stock. If we know the old stock works well, this also acts as a suitable positive control.<br>Active Motif offers a wide variety of high quality antibodies for studying histone and DNA modifications, transcription factors, nuclear receptors, and more. Our antibodies are manufactured in-house, where they undergo rigorous developmental and validating procedures to ensure their quality and performance. These antibodies are validated for use in many applications, such as chromatin immunoprecipitation (ChIP), ChIP-Seq, immunoblot, and immunofluorescence.

Eukaryotic cell lines

Policy information about cell lines

Cell line source(s)	Rat H9c2 and mouse C2C12 cells were all purchased from the ATCC (Manassas, VA, USA).
Authentication	The cell lines were authenticated by the vendor. We did not perform any validation except for visual evaluation of morphology.
Mycoplasma contamination	These cell lines were tested negative for mycoplasma contamination.
Commonly misidentified lines<br>(See ICLAC register)	No misidentified cell lines were used in the study.

Animals and other organisms

Policy information about studies involving animals; ARRIVE guidelines recommended for reporting animal research

Laboratory animals	All mice were maintained on standard chow diet at a constant temperature of 23 °C and humidity of 50% under an artificial 12 h light and 12 h dark cycle with ad libitum access to water in the SPF animal house. Cre/lox system was employed to achieve tissue-specific transgenic mice. Sdhaf4 floxed mice (homozygous referred as Sdhaf4fl/fl, heterozygous referred as Sdhaf4fl/-) were generated by Beijing Biocytogen Co. Ltd. Briefly, two sgRNAs were designed to generate a ~3kb chromosomal deletion (exon 1-2) at the Sdhaf4 locus in the mouse genome. All the mice were in C57BL/6J background and backcrossed at least 7 generations. Ckmm-cre (No. 006475) mice and Myh6-cre/Ers1* (No. 005657) were purchased from Jackson Laboratory. Ckmm-cre were crossed with Sdhaf4fl/fl mice to generate skeletal and cardiac muscle specific knockout mice (Sdhaf4fl/fl, Ckmm-Cre, Sahaf4fl/-, Ckmm-Cre referred as homozygous and heterozygous knockout). The littermates Sdhaf4fl/fl mice were used as control group. Similarly, cardiac myocytes specific knockout mice were achieved through crossing Sdhaf4fl/fl mice with Myh6-cre mice. Homologous recombination was achieved by intraperitoneally injecting of tamoxifen at 8 weeks old. The littermates Sdhaf4fl/fl, Mer-CreMer mice which were intraperitoneally injected with corn oil were used as control. For the intervention study, Sahaf4fl/fl, Ckmm-Cre mice were daily injected with Mdivi-1 (Sigma-Aldrich, M0199) dissolved in DMSO at the dose of 50 mg/kg bodyweight, and Sdhaf4fl/fl, Ckmm-Cre mice injected with DMSO were used as control. The supplement of sodium fumarate dibasic (Sigma-Aldrich, F1506) to Sdhaf4fl/fl, Ckmm-Cre mice were through drinking water at the dose of 2 g/L. All the treatments were started at age of 4 weeks old, physiological and biochemical indicators were detected after 3 weeks of treatment. Survival rates were calculated through continuous supplement, both male and female. Echocardiography was used to detect the control, heterozygous knockout and homozygous knockout mice at 8 weeks old of CKMM-Flox mice. Male mice aged 3 weeks or 8 weeks were used for molecular biological analysis and omics analysis. For the MI mode, all mice were used C57BL/6 male at 8 weeks old to surgery.
Wild animals	No wild animals were used in the study.
Field-collected samples	No field-collected samples were used in the study.
Ethics oversight	For animal study, the protocol was approved by the Animal Care and Use Committee of the School of Life Science and Technology, Xi'an Jiaotong University (No. 2017-0025). All procedures were performed in accordance with the United States Public Health Services Guide for the Care and Use of Laboratory Animals, and all possible efforts were made to minimize the stress and number of animals utilized in this study.

Note that full information on the approval of the study protocol must also be provided in the manuscript.
