# Supplementary material for: Cardiac disruption of SDHAF4-mediated mitochondrial complex II assembly promotes dilated cardiomyopathy
Source: Nat Commun. 2022 Jul 8;13:3947. doi: 10.1038/s41467-022-31548-1 (PMC9270418; doi:10.1038/s41467-022-31548-1)
Supplement: Supplementary file 3 — Supplementary Information [file 41467_2022_31548_MOESM3_ESM.pdf]

Supplementary Information: Cardiac disruption of SDHAF4-mediated mitochondrial complex II assembly promotes dilated cardiomyopathy

Z. Feng et al.

Supplementary Figure 1

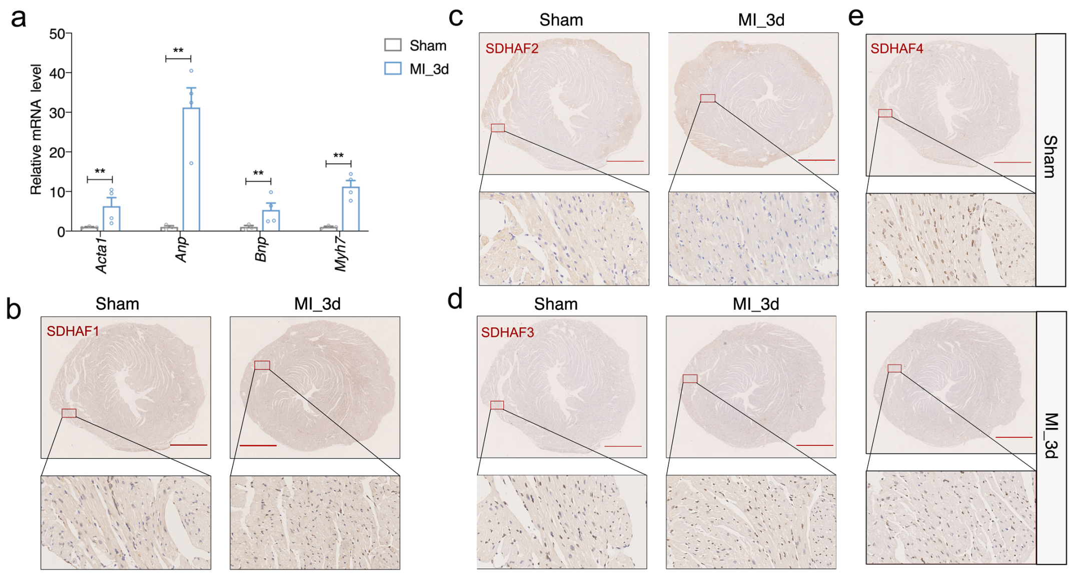

Supplementary Figure 1. Downregulated SDHAF4 expression in myocardial infarction (MI) mice. a, Relative expression of hypertrophic and fibrotic associated genes in control and MI mice at day 3 after the surgery (Sham, n=3; MI_3d, n=4, *P*<0.0001). **b-e**, Immunohistochemistry staining of SDHAF1/2/3/4 in the control and MI mice at day 3 after the surgery, scale bar, 3 mm. Values are mean ± SEM, ***P*< 0.01. Statistical significance was determined by two-tailed Student's *t*-test (**a**). Source data are provided in Source Data file.

Supplementary Figure 2

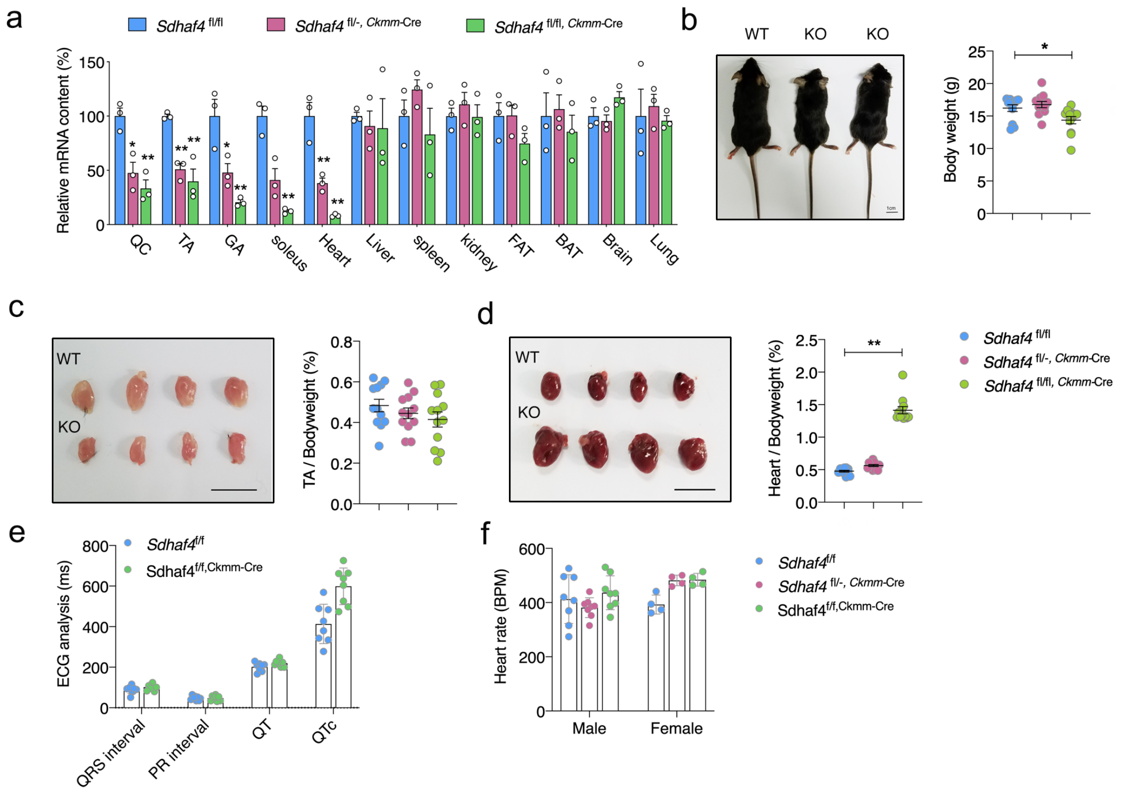

Supplementary Figure 2. Characterization of muscle *Sdhaf4* knockout mice. a, mRNA levels of *Sdhaf4* in tissues of *Sdhaf4*$^{fl/fl}$ (WT), heterozygous KO (*Sdhaf4*$^{fl/-, Ckmm\text{-}Cre}$), and homozygous mutant (*Sdhaf4*$^{fl/fl, Ckmm\text{-}Cre}$, or KO) mice, n=3, *P*=0.013, 0.003, 0.001, 0.006, 0.007, 0.013, 0.0006, 0.009, 0.001. **b-d**, Body weight, ratio of tibialis anterior (TA) muscle and heart weight (*P*=0.02) of mice at 8 weeks old, n=12, image showing only WT and homozygous KO mice, scale bar, 1 cm. **e**, ECG analysis of WT, homozygous *Sdhaf4* KO mice at the age of 8 weeks (QRS interval, PR interval, QT, and QTc), n=8. **f**, Heart rate of WT, heterozygous, and homozygous *Sdhaf4* KO mice during echocardiography study, n=8. Values are mean ± SEM, *$P< 0.05$, **$P< 0.01$. Statistical significance was determined by two-tailed Student's *t*-test (**a**). Source data are provided in Source Data file. QC: quadriceps; GA: gastrocnemius; TA: tibia anterior.

Supplementary Figure 3

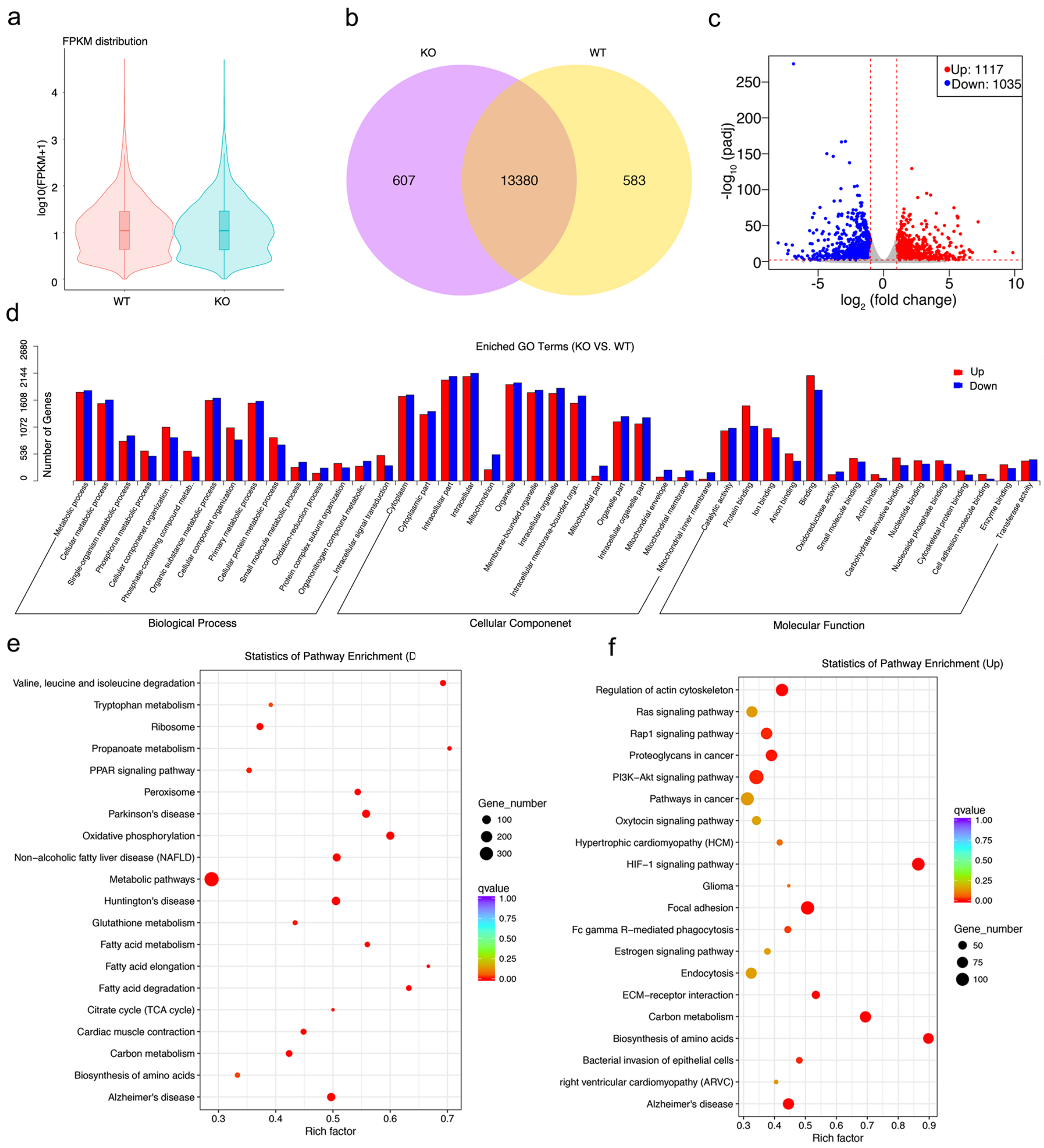

Supplementary Figure 3. RNA-sequencing analysis of WT and *Sdhaf4* mutant (*Sdhaf4*$^{fl/fl,\ Ckmm-Cre}$, or KO) mice. a-f, RNA-sequencing analysis of left ventricles of WT and *Sdhaf4*-KO mice: Violin plot representing distribution of FPKM values, the five characteristic values indicate maximum, upper quartile, mid-value, lower quartile, and minimum, respectively (**a**); Venn diagram analysis(**b**); Volcano plot (**c**); The top 15 enriched GO terms of biological process, cellular

component and molecular function (**d**); Scatterplot of KEGG analysis representing top 20 down regulated pathway (**e**); Scatterplot of KEGG analysis representing top 20 up regulated pathway (**f**), n=6.

Supplementary Figure 4

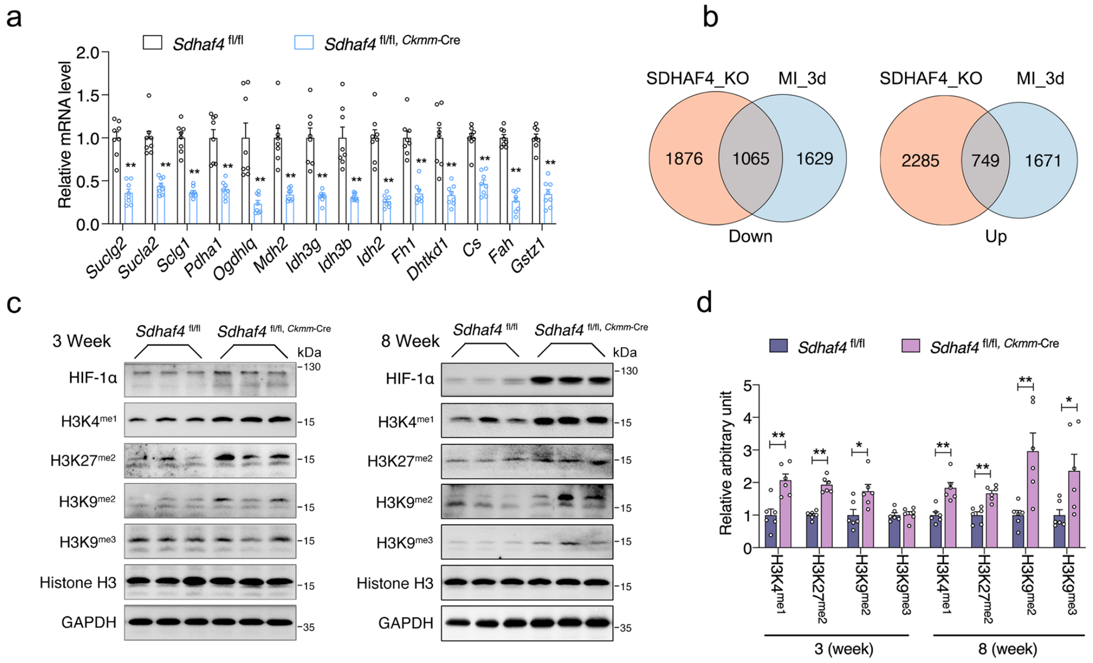

Supplementary Figure 4. Cardiac loss of *Sdhaf4* induces dramatic alternations of gene expression. a, qPCR analysis of mitochondrial metabolism associated genes in left ventricle of WT and *Sdhaf4* mutant (*Sdhaf4*$^{fl/fl,\ Ckmm\text{-}Cre}$, KO), n=8, *P*<0.0001. **b**, Venn diagram analysis of RNA expression pattern in the heart tissues of *Sdhaf4*-KO mice and day 3 MI mice. **c-d,** Immunoblots for histone methylation level of WT and muscle *Sdhaf4*-KO mice at the age of 3 and 8 weeks (**c**, representative blotting image; **d**, summary analysis of arbitrary unit, n=6, *P*=0.002, 0.00001, 0.02, 0.001, 0.0006, 0.006, 0.02). Values are mean ± SEM, **P*< 0.05, ***P*< 0.01. Statistical significance was determined by two-tailed Student's *t*-test (**a**, **d**). Source data are provided in Source Data file.

Supplementary Figure 5

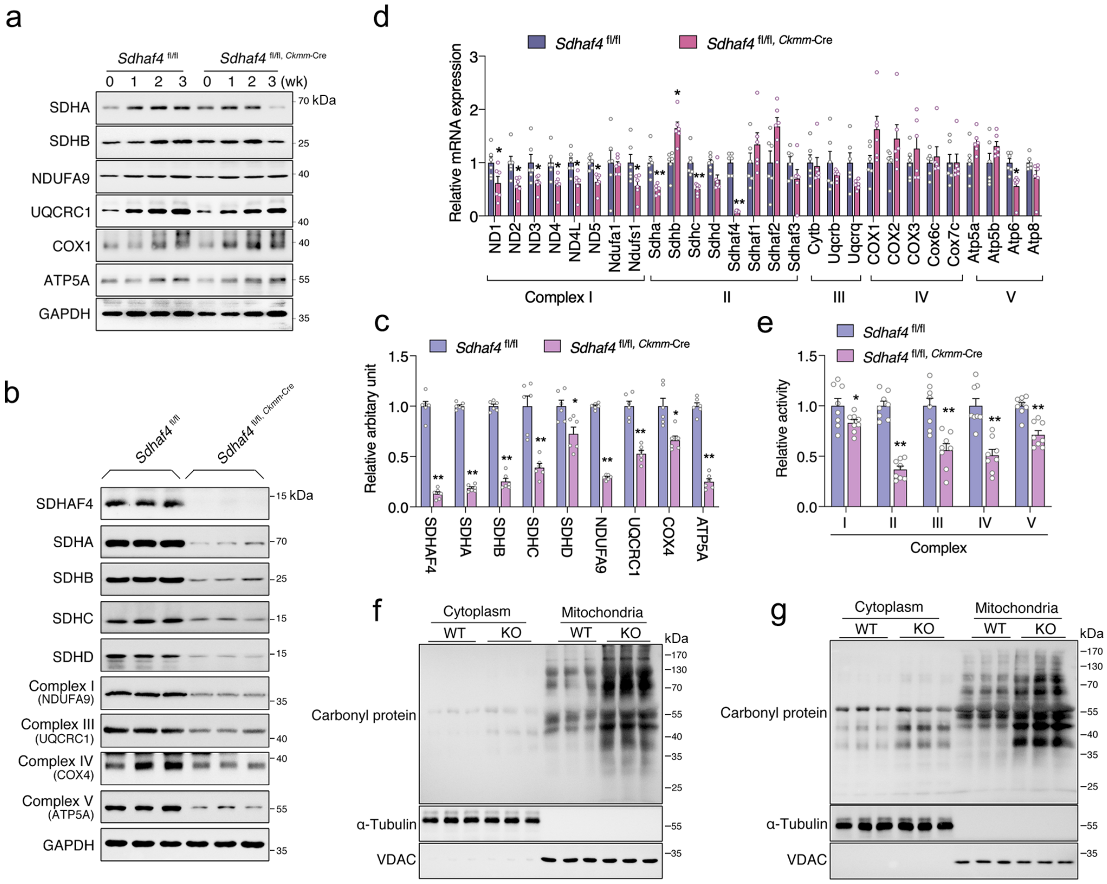

Supplementary Figure 5. Loss of *Sdhaf4* disrupts complex II assembly and promotes mitochondrial dysfunction. a, Immunoblots for mitochondrial complex subunits in left ventricles of WT and *Sdhaf4* mutant (*Sdhaf4*$^{\text{fl/fl, }Ckmm\text{-Cre}}$, KO) at the postnatal age 1, 2, and 3 weeks respectively (n = 3 biologically independent mice per group). **b-c**, Immunoblots for SDHAF4 and mitochondrial complex subunits in left ventricles of WT and muscle *Sdhaf4*-KO mice at age of eight weeks, representative blotting images, (**b**), summary analysis of arbitrary unit (**c,** *P*<0.0001, *P*=0.011, 0.02), n=6. **d**, mRNA levels of mitochondrial complex subunits in left ventricles of WT and muscle *Sdhaf4*-KO mice (n=6, *P*=0.04, 0.011, 0.04, 0.014, 0.011, 0.03, 0.018, 0.0014, 0.012, 0.0012, 0.0001, 0.04). **e**, Analysis of complex activities of mitochondrion from left ventricles WT and muscle *Sdhaf4*-KO mice at age of eight weeks, n=6, *P*<0.0001, *P*=0.04. **f-g**, Immunoblots for

carbonyl proteins of left ventricles from WT and muscle *Sdhaf4*-KO mice at age of three (**f**) and eight (**g**) weeks respectively (n = 3 biologically independent mice per group). Values are mean ± SEM, *$P < 0.05$, **$P < 0.01$. Statistical significance was determined by two-tailed Student's *t*-test (**c-e**). Source data are provided in Source Data file.

Supplementary Figure 6

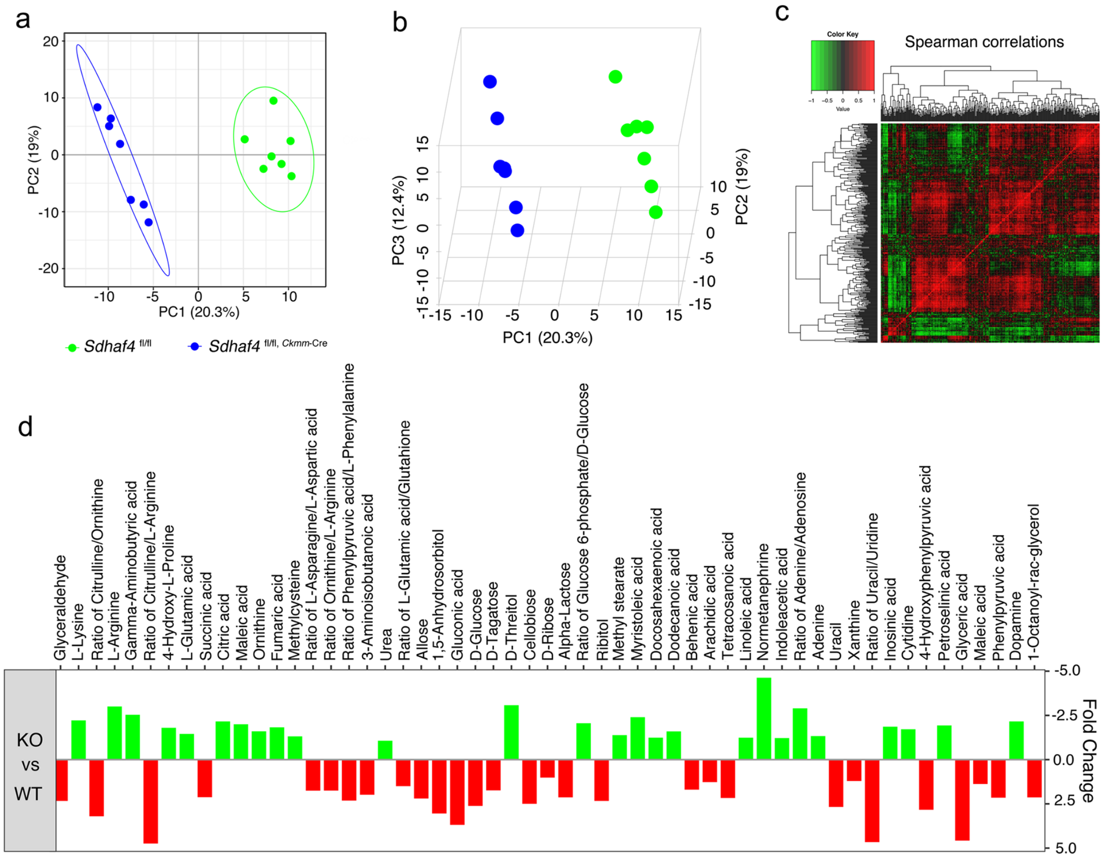

Supplementary Figure 6. Metabolomics analysis for hearts. The untargeted metabolomics profiling was performed with left ventricles of WT and muscle *Sdhaf4* mutant(*Sdhaf4*$^{\text{fl/fl}, \textit{Ckmm}\text{-Cre}}$) at age of 8 weeks. **a**, 2D PLS-DA scores plot revealing classifications of the samples, n=7. **b**, 3D PLS-DA scores plot revealing classifications of the samples, n=7. **c**, Pearson correlation map depicting the overall metabolite relations among samples, n=7. **d**, The differential metabolite profiles between groups, n=7.

Supplementary Figure 7

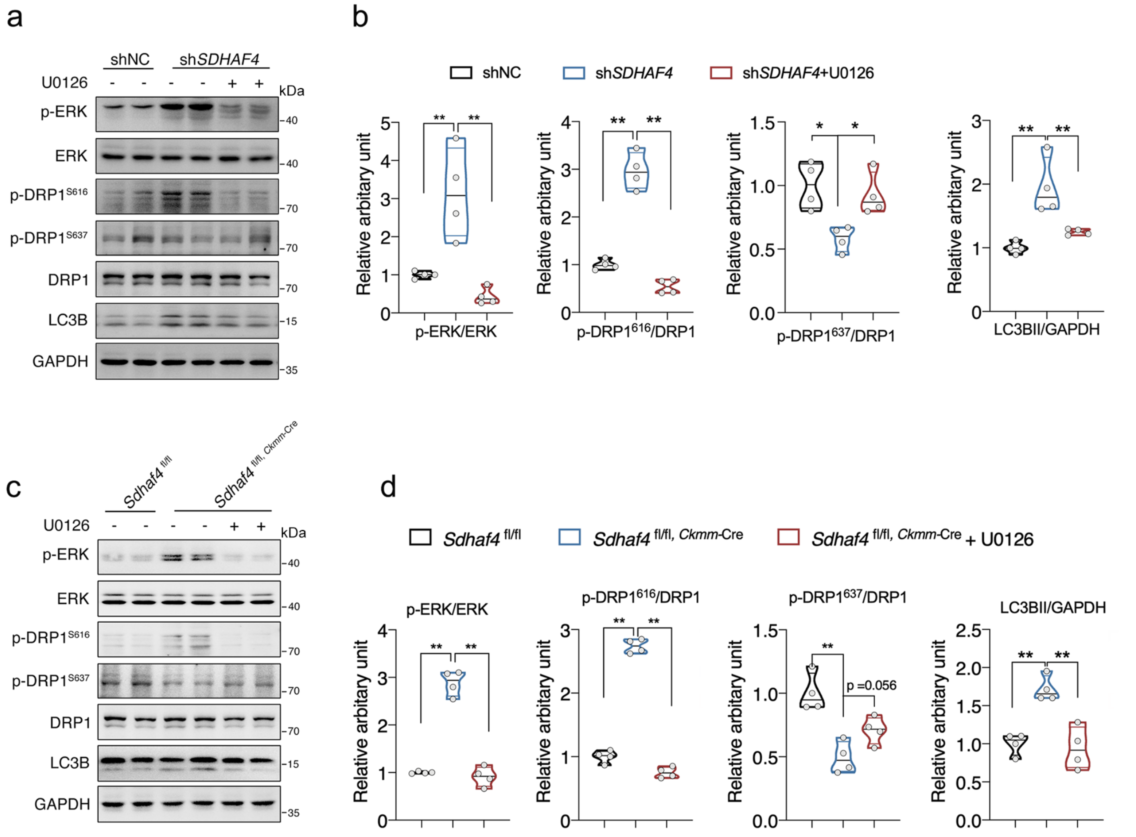

Supplementary Figure 7. Loss of SDHAF4 promotes mitophagy via phosphorylation of ERK. a-b, Immunoblots for p-ERK, p-DRP1^{616}, p-DRP^{637}, and LC3B in sh*SDHAF4* lentivirus infected H9c2 cells with or without U0126 treatment, representative blotting image (**a**), summary analysis of arbitrary unit (**b,** *P*<0.0001, *P*=0.023, 0.03), n=4. **c-d**, Immunoblots for p-ERK, p-DRP1^{616}, p-DRP^{637}, and LC3B for left ventricles of hearts from WT, *Sdhaf4* mutant(*Sdhaf4*$^{\text{fl/fl, } Ckmm\text{-Cre}}$) with or without U0126 treatment for two weeks, representive blotting image (**c**), summary analysis of arbitrary unit (**d,** *P*<0.0001), n=4. Values are mean ± SEM, **P*< 0.05, ***P*< 0.01. Statistical significance was determined by two-tailed Student's *t*-test (**c-e**). Source data are provided in Source Data file.

Supplementary Figure 8

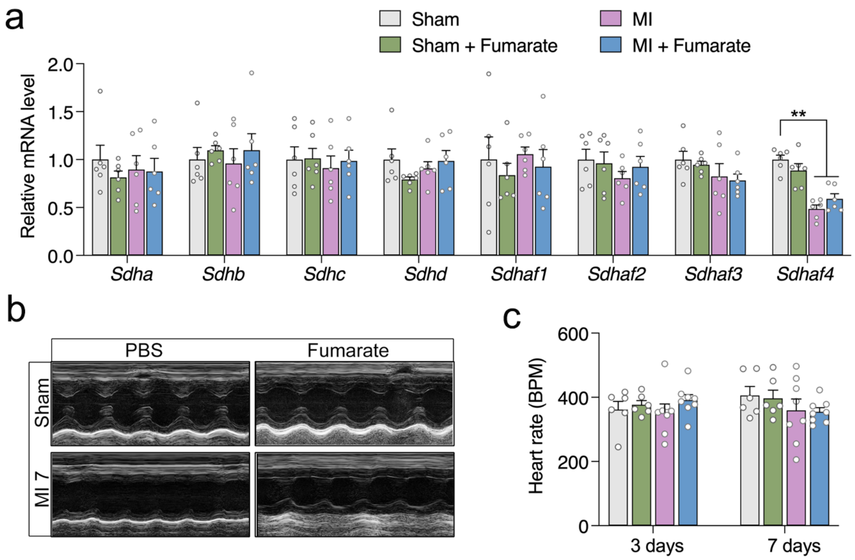

Supplementary Figure 8. Fumarate supplement improves cardiac function in MI mice. a, Relative expressions of *Sdhs* and *Sdhafs* in left ventricle of control and MI mice with or without fumarate supplement for 7 days, n=6, P<0.0001. **b-c**, Heart function measurements of mice: representative M-mode echocardiography (**b**), heart rate (**c**) (n=6 for Sham and Sham + Fumarate groups, n=8 for MI and MI + Fumarate groups). Values are mean ± SEM, *P< 0.05, **P< 0.01. Statistical significance was determined by two-tailed Student's *t*-test (**c-e**). Source data are provided in Source Data file.

Uncropped blots for Supplementary Figure 4

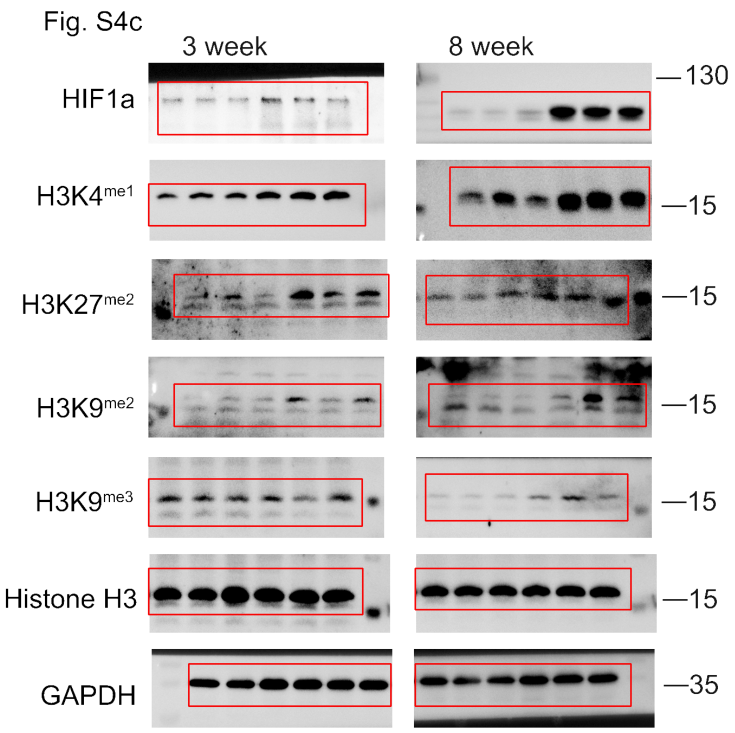

Uncropped blots for Supplementary Figure 5

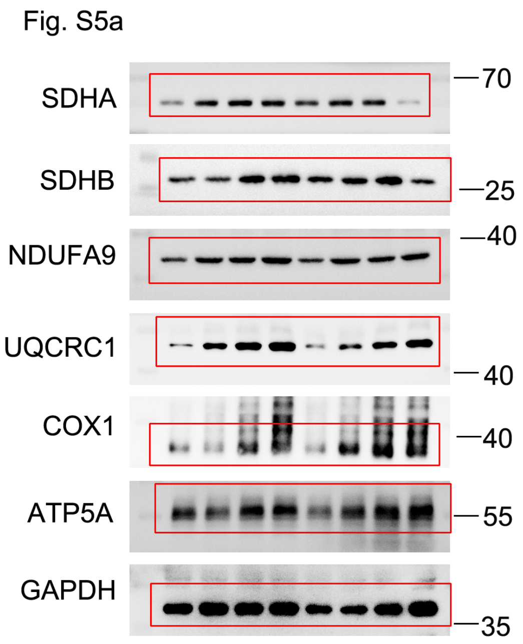

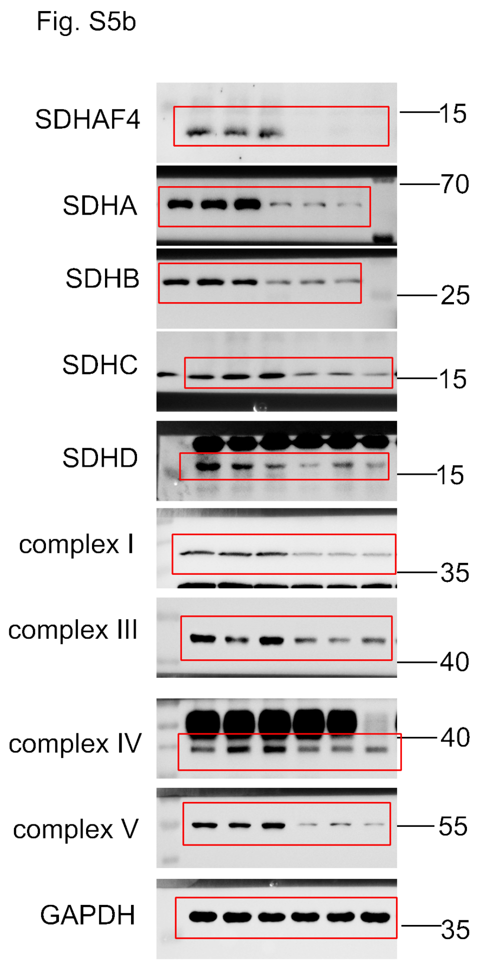

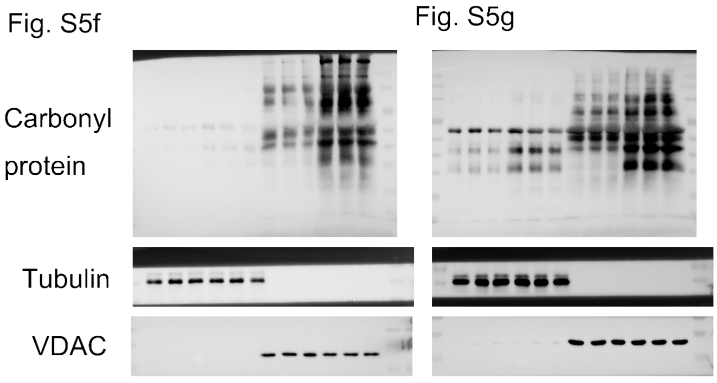

Uncropped blots for Supplementary Figure 7

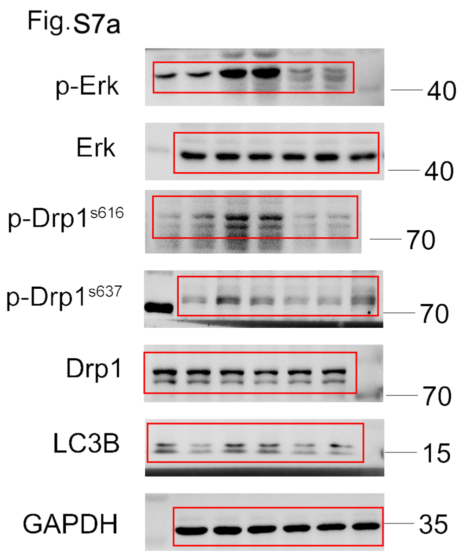

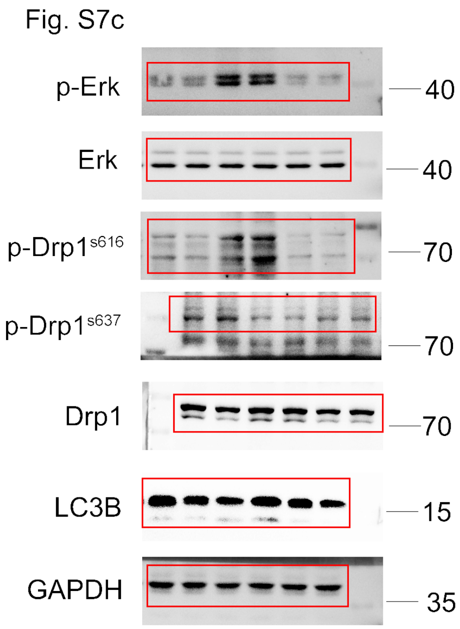

Supplemental Table 1

TCA cycle-related genes consistently downregulated in MI, DCM, and *Sdhaf4*-KO hearts

Gene	Description
Cs	citrate synthase, mitochondrial precursor
Fh1	fumarate hydratase, mitochondrial precursor
Idh2	isocitrate dehydrogenase [NADP], mitochondrial
Idh3a	isocitrate dehydrogenase [NAD] subunit alpha
Idh3b	isocitrate dehydrogenase 3, beta subuni
Mdh2	malate dehydrogenase, mitochondrial precursor
Ogdhl	2-oxoglutarate dehydrogenase-like
Pdha1	pyruvate dehydrogenase E1 component subunit
Sucla2	Mus musculus adult male cerebellum cDNA, RIKEN full-length enriched library, clone:1500017C15 product:succinate-Coenzyme A ligase, ADP-forming, beta subunit, full insert sequence.
Suclg1	succinyl-CoA ligase [GDP-forming] subunit alpha
Suclg2	succinyl-CoA ligase [GDP-forming] subunit beta
