# Supplementary material for: Cardiac disruption of SDHAF4-mediated mitochondrial complex II assembly promotes dilated cardiomyopathy
Source: Nat Commun. 2022 Jul 8;13:3947. doi: 10.1038/s41467-022-31548-1 (PMC9270418; doi:10.1038/s41467-022-31548-1)
Supplement: Supplementary file 4 — Description of Additional Supplementary Files [file 41467_2022_31548_MOESM4_ESM.pdf]

Description of Additional Supplementary files

File name: Supplementary data 1
Description: All the primer sequences used in real-time PCR assay of this study.

File name: Supplementary data 2
Description: Raw data for metabolomics study. Metabolites in the study samples were annotated with mammalian metabolite database *JiaLib*^{TM} using a strict matching algorithm incorporated in XploreMET software (Version 2.0) that used both retention times and fragmentation patterns in the mass spectrum. There are total of 363 metabolites detected, while only 135 metabolites annotated, the remaining unknowns that do not match the reference library.
